# Fructose-Containing Dietary Exposures and Pediatric Atopic Disease: A Review of Epidemiologic Evidence

**DOI:** 10.3390/nu18071057

**Published:** 2026-03-26

**Authors:** Charles Prendergast, Kamil Barański

**Affiliations:** Department of Epidemiology, Faculty of Medical Sciences in Katowice, Medical University of Silesia in Katowice, 40-751 Katowice, Poland

**Keywords:** fructose, sugar-sweetened beverages, asthma, atopy, children, asthmatic tendency

## Abstract

**Background**: Mechanistic evidence increasingly implicates fructose exposures as contributors to the development and exacerbation of asthma and other atopic diseases. Proposed mechanisms include gut dysbiosis, impaired epithelial barrier integrity in the gut and airways, metabolic endotoxemia, and amplification of type 2 immune responses. However, epidemiologic findings linking fructose intake with asthma and atopic disorders remain heterogeneous. **Objective**: To conduct a review of epidemiologic studies evaluating associations between dietary fructose-containing exposures and atopic outcomes in pediatric populations. **Methods**: A systematic search of PubMed and Embase identified cohort, case-control, cross-sectional, and randomized feeding studies assessing fructose exposure in relation to asthma and atopic outcomes in pediatric populations. Eligibility screening, data extraction, and risk-of-bias assessment were conducted by one reviewer and confirmed by the other. **Results**: Seventeen epidemiologic studies met criteria. Multiple cohorts (e.g., BRISA, PIAMA) reported modest to moderate associations between higher sugar-sweetened beverage (SSB) intake and pediatric asthma or “asthma traits.” Cross-sectional analyses from NHANES and the National Children’s Study showed stronger associations, with greater fructose exposures linked to two- to five-fold higher odds of asthma. High fructose beverage consumption demonstrated the most consistent positive associations. Large ISAAC-based studies reported largely null findings, reflecting broad dietary exposure categories and limited specificity for fructose-rich beverages. Evidence for rhinitis, eczema, and sensitization was directionally consistent. **Conclusions**: Despite heterogeneity, the convergence of mechanistic plausibility with epidemiologic signals supports a potential contributory role of high fructose exposure in pediatric atopic disease. More rigorous longitudinal studies with biomarker-based exposure assessment are needed to refine causal inference.

## 1. Introduction

Atopic diseases, including asthma, allergic rhinitis, and eczema, constitute a major and growing global public health burden [1]. In 2021, an estimated 260 million people had asthma, and 129 million had atopic dermatitis, with rhinitis affecting roughly one-quarter to one-third of the global population [2]. In childhood, these conditions represent some of the most common chronic disorders, with evidence of rising prevalence and marked regional variability [3,4,5,6]. Recent epidemiological data show continued increases in pediatric allergic diseases across Europe and globally [7,8], with asthma remaining the most prevalent chronic condition in children and eczema and rhinitis also demonstrating sustained growth [7,8,9].

Long-term surveillance through ISAAC and the Global Asthma Network documents increasing prevalence, symptom burden, and quality-of-life impairment across multiple regions, particularly Europe and the Americas [1,10]. These data reveal substantial socioeconomic disparities, with higher asthma prevalence in children from lower socioeconomic backgrounds [1]. Although genetic predisposition underlies susceptibility, the rapid rise in atopic diseases underscores the importance of environmental and lifestyle factors. Increasing evidence suggests that gene–environment interactions and epigenetic mechanisms may play an important role in mediating the effects of environmental exposures on immune development and allergic disease susceptibility [11].

### 1.1. Diet as a Modifiable Risk Factor

Dietary patterns are increasingly recognized as key environmental determinants of immune development and allergic disease risk [12]. Westernized diets, characterized by high intakes of ultra-processed foods, refined carbohydrates, and SSBs, promote pro-inflammatory states and epithelial barrier dysfunction [13,14]. In Central and Eastern Europe, including Poland, urbanization and food marketing have accelerated the adoption of these dietary patterns [15]. National reports show that Polish children frequently exceed WHO recommendations for free sugar intake, primarily through sweets and carbonated beverages [15,16]. Fructose-containing sugars, in particular, have gained attention due to their distinct metabolic properties and biologically plausible links to allergic disease via effects on gut integrity, inflammation, and hepatic metabolic signaling. Emerging evidence also highlights the importance of critical developmental windows, particularly the prenatal and early postnatal periods, during which environmental exposures may have disproportionate effects on immune maturation and allergy susceptibility. Early-life dietary exposures during these sensitive windows may therefore play a particularly important role in shaping long-term risk of atopic disease.

### 1.2. Fructose and the Gut–Immune–Lung Axis

Fructose absorption is variable and saturable; unabsorbed fructose reaches the colon [17,18], although chronic high intake upregulates GLUT5 and enhances absorption [19]. Colonic fructose undergoes rapid bacterial fermentation, altering microbiota composition, reducing short-chain fatty acids, and increasing luminal osmotic load [20]. These changes impair tight-junction integrity, increase intestinal permeability, and facilitate translocation of microbial products such as LPS into circulation [20]. Resulting metabolic endotoxemia activates pattern-recognition receptors, including TLR4, driving cytokine release and systemic immune activation [21]. Through the Gut–Immune–Lung Axis, a pathway implicated in asthma and allergic diseases [22,23], fructose-mediated dysbiosis and barrier dysfunction enhance type 2 inflammatory priming by promoting airway epithelial alarmin release (e.g., IL-33) [24], dendritic-cell activation [25], and mast-cell and basophil responsiveness [26]. Reduced SCFA availability further impairs Treg differentiation and promotes Th2 polarization [27].

### 1.3. Fructose and the Gut–Liver Axis

Fructose is primarily metabolized in the liver, where it is rapidly phosphorylated by ketohexokinase to fructose-1-phosphate and cleaved to triose phosphates that enter glycolysis downstream of phosphofructokinase-1, bypassing a major regulatory checkpoint. This unregulated flux favors gluconeogenesis and de novo lipogenesis, generating substrates for triacylglycerol synthesis and VLDL export [28]. Excess acetyl-CoA is converted to acetate and medium-chain fatty acids, metabolites recently linked to asthma pathophysiology [29]. Rapid fructose phosphorylation also depletes ATP and increases uric acid production, a pro-inflammatory DAMP that activates hepatic NLRP3 inflammasomes and IL-1β and IL-6 secretion [25,30]. Concurrent lipotoxicity and oxidative stress propagate systemic low-grade inflammation that affects peripheral immune tissues, including the lung [31,32]. Fructose metabolism shares key biochemical features with ethanol, including bypass of regulatory pathways, enhanced lipogenesis, uric acid elevation, and mitochondrial stress, suggesting that the pediatric liver may be particularly vulnerable [33].

### 1.4. Fructose as an Adipogenic Driver

Fructose is strongly adipogenic due to its preferential routing toward lipogenesis rather than regulated energy production. Unrestricted triose phosphate flux increases acetyl-CoA and malonyl-CoA, promoting triglyceride synthesis while inhibiting fatty acid oxidation [28,30]. These processes, compounded by uric-acid-driven inflammation and impaired insulin signaling, promote adipocyte hypertrophy and ectopic fat deposition [28]. Given the established relationship between childhood adiposity and asthma severity—“obesity-related asthma” [34]—as well as higher rates of other atopic diseases in children with elevated adiposity [35,36], fructose-driven weight gain provides an additional indirect pathway linking fructose exposure to atopic disease.

### 1.5. A Review of the Epidemiological Evidence

Despite strong mechanistic plausibility, epidemiological studies examining fructose-rich dietary exposures (e.g., free sugars, SSBs) and pediatric atopic outcomes report heterogeneous findings. While recent reviews have specifically examined fructose-rich beverage consumption and childhood asthma [16], broader evidence across atopic diseases remains unsynthesized. This review synthesizes the available evidence to clarify the consistency, magnitude, and methodological quality of observed associations.

## 2. Materials and Methods

### 2.1. Objective and Eligibility Criteria

This study was conducted as a rapid review, defined as a form of evidence synthesis that streamlines elements of the systematic review process to produce evidence within a shorter time frame. In the present review, streamlining included restriction to two bibliographic databases, use of single-reviewer screening/data extraction with verification by a second reviewer rather than a fully independent duplicate assessment, and completion of the review within a condensed time frame.

This review aimed to evaluate epidemiologic evidence assessing the association between dietary fructose-containing sugars and pediatric atopic outcomes. Eligible studies included original human research involving children aged 0–18 years that examined associations between dietary fructose, added sugars, SSBs, fruit juice, high fructose corn syrup (HFCS), or excess free fructose and atopic outcomes. Eligible epidemiologic study designs were cohort, case-control, and cross-sectional studies, as well as randomized controlled feeding trials with human participants.

Comparators included lower or no fructose intake, alternative sugar exposures, or unexposed groups. Eligible outcomes were incident or prevalent asthma, wheeze, allergic rhinitis, atopic dermatitis, food allergy, and IgE sensitization.

Excluded were mechanistic animal or in vitro studies, review articles, meta-analyses, commentaries, case reports, non-systematic opinion pieces, and studies that did not report an epidemiologic estimate. No language restrictions were applied at the search stage.

### 2.2. Search Strategy and Data Sources

A systematic search was conducted in PubMed and Embase on 20 November 2025 and restricted to studies published from January 2010 onward to capture contemporary dietary exposures, food formulations, and atopic disease definitions. Screening, data extraction, and risk-of-bias assessment using the Newcastle–Ottawa Scale were conducted by one reviewer and independently verified by a second reviewer. Domain-level Newcastle–Ottawa Scale assessments for each individual-level observational study are provided in Supplementary Table S2 for transparency of the risk-of-bias evaluation.

PubMed and Embase were selected because, in combination, they provide comprehensive, curated coverage of biomedical, pediatric, respiratory, and nutritional epidemiology, including the primary journals publishing research on fructose-containing beverages and pediatric asthma. Together, these databases capture most relevant observational studies while minimizing redundancy across overlapping indexing systems. These databases also capture a substantial proportion of the biomedical literature relevant to nutritional epidemiology and pediatric respiratory disease, although restricting the search to two databases may have reduced the sensitivity of the search strategy.

This review was conducted as a rapid review using systematic search criteria with narrative synthesis designed to characterize the epidemiologic evidence linking fructose-containing dietary exposures with pediatric atopic outcomes. The review applied predefined eligibility criteria, structured literature searches, standardized data extraction, and formal risk-of-bias assessment. The review protocol was not prospectively registered in PROSPERO or another registry, which may limit external verification of prespecified methods. However, full search strategies, screening decisions, and the PRISMA flow diagram are provided in the Supplementary Materials to improve transparency. The review was conducted and reported with reference to the PRISMA 2020 reporting guidelines. A completed PRISMA 2020 checklist indicating the location of each reporting item within the manuscript is provided as Supplementary Table S3.

### 2.3. Literature Search Strategy

A comprehensive literature search was conducted to identify studies examining the association between fructose-containing beverages, sugar-sweetened beverages, and atopic outcomes in pediatric populations. Searches were performed in PubMed and Embase from January 2010 to November 2025, restricted to human studies.

In PubMed, the search strategy combined controlled vocabulary (MeSH terms) and free-text keywords for three main concepts: (1) dietary exposures, (2) atopic outcomes, and (3) pediatric populations. Exposure-related terms included fructose, high fructose corn syrup, sugar-sweetened beverages, soft drinks, sucrose, added sugars, and fruit juice. Outcome-related terms included asthma, wheeze, atopic dermatitis/eczema, allergic rhinitis, food allergy, atopy, and immunoglobulin E (IgE). Population-related terms included infant, child, toddler, and adolescent. The PubMed animal-only filter (animals[mh] NOT humans[mh]) was applied to exclude non-human studies.

In Embase, an analogous strategy was implemented using Emtree terms and title/abstract keywords for fructose-containing beverages, atopic and allergic outcomes, and pediatric populations. The Embase search was similarly limited to human studies published between 2010 and 2025. Full electronic search strategies for each database are provided in Supplementary Table S1.

In addition, the reference lists of all included studies and relevant review articles were hand-searched to identify any additional eligible primary epidemiologic studies not captured by the database searches.

### 2.4. Study Selection

All search results were exported and deduplicated across databases. Title/abstract screening, full-text review, data extraction, and risk-of-bias assessment were conducted by one reviewer and verified in full by a second independent reviewer. All decisions received dual scrutiny, with any discrepancies resolved through collaborative discussion to ensure consensus and methodological robustness. This comprehensive sequential verification approach provided high reliability across all review stages.

**Figure 1:**
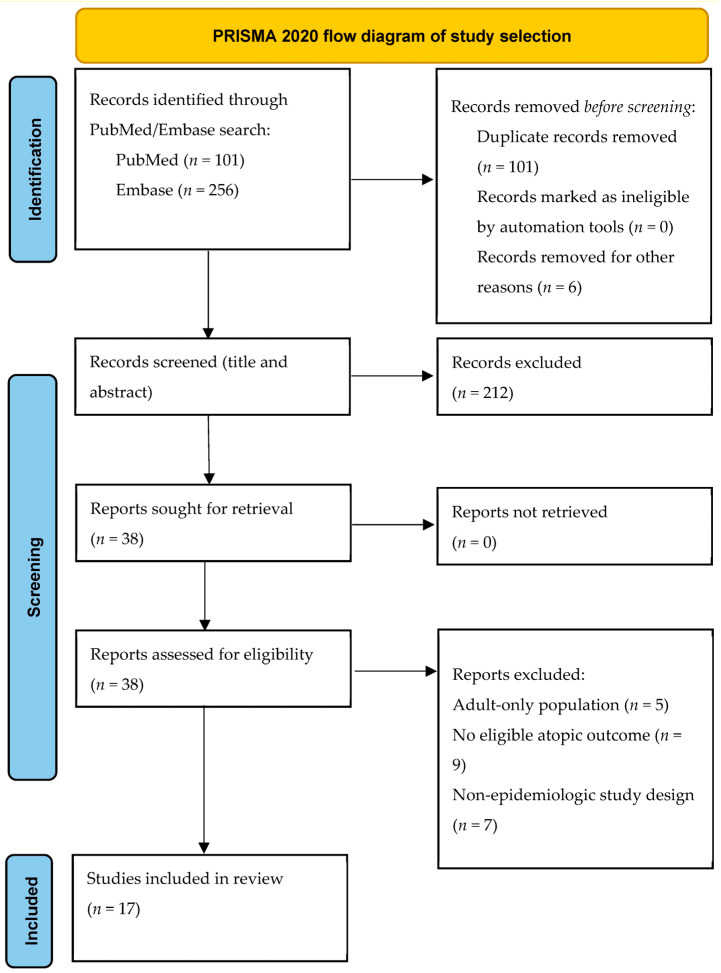
PRISMA 2020 flow diagram of study selection.

Studies were excluded if they: (1) lacked an epidemiologic study design; (2) did not assess fructose-containing sugars as an exposure; (3) did not report an atopic outcome; (4) involved adults only; or (5) were review articles, commentaries, mechanistic studies, ecological studies, or case reports. Reasons for exclusion at the full-text stage were recorded. The study selection process is summarized in a PRISMA flow diagram (Figure 1).

### 2.5. Data Extraction

A standardized extraction form captured the following variables from each included study: study design and setting; sample size and population characteristics; exposure type (e.g., fructose, HFCS, SSBs, fruit juice, and added sugars); exposure assessment method; outcome definition and ascertainment; statistical models and covariates; effect estimates (odds ratios, risk ratios, and hazard ratios) and 95% confidence intervals. One reviewer independently extracted data, while the other confirmed the appropriateness. Discrepancies were resolved by consensus.

For graphical synthesis, studies reporting adjusted odds ratios with corresponding 95% confidence intervals were displayed in a forest plot. Because of substantial heterogeneity in study design, exposure definitions, and outcome ascertainment, no quantitative pooling or meta-analysis was performed. The forest plot was used solely to display the range, direction, and dispersion of individual study estimates and to support narrative synthesis.

### 2.6. Quality Assessment

The risk of bias for observational studies was assessed using the Newcastle–Ottawa Scale (NOS), evaluating participant selection, exposure assessment, outcome ascertainment, and control for confounding. Assessments were conducted by one reviewer and independently verified by a second reviewer, with discrepancies resolved by consensus.

### 2.7. Data Synthesis

Due to heterogeneity in exposure definitions, outcome measures, and study designs, a meta-analysis was not performed. The findings were synthesized narratively, with effect estimates summarized in tables. Where possible, the results were grouped by exposure type (e.g., SSBs, fructose, and fruit juice) and by outcome domain (asthma/wheeze, atopic dermatitis, allergic rhinitis, food allergy, IgE).

Patterns, inconsistencies, and potential sources of heterogeneity were described, and evidence gaps were highlighted.

## 3. Results

Seventeen epidemiologic analyses met inclusion criteria, comprising four longitudinal birth cohorts (BRISA, Project Viva, PIAMA, and Emerson puberty cohort), and thirteen cross-sectional or case–control studies from national surveys and clinic samples (including NHANES, National Children’s Study, ISAAC Phase Two, French Six Cities, California Health Interview Survey, PeNSE, Korean Youth Risk Behavior Survey, and Norwegian and Brazilian clinic-based studies). Most were large, population-based cohorts or nationally representative surveys (*n* = 400 to >865,000), although several smaller clinic-based and one very small puberty cohort (*n* = 17) were included (Table 1).

Exposures primarily comprised SSBs, fruit juices, and excess free fructose–rich beverages (HFCS-sweetened soda, fruit drinks, and apple juice), assessed using dietary recalls or food-frequency instruments. Outcomes commonly included current asthma, wheeze, or asthma traits, with rhinitis, eczema, sensitization, and severity examined less frequently. Multivariable models adjusted for major confounders (age, sex, socioeconomic status, adiposity, smoking, and sometimes broader dietary or maternal factors). Effect estimates ranged from null to modest increases in asthma risk, with larger odds ratios in analyses focused on high-EFF beverages or very high soda intake. Overall, the study quality was moderate, with stronger inference from prospective cohorts and high-quality survey analyses, and greater limitations in small clinic-based designs.

**Figure 2:**
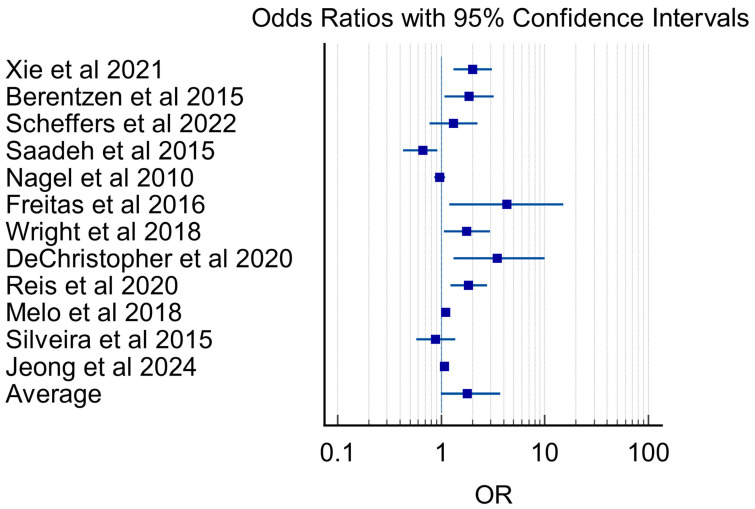
Forest plot of study-specific odds ratios for fructose-containing beverage exposures and atopic outcomes with 95% confidence intervals. Only studies reporting odds ratios were included; no meta-analysis or pooled summary estimate of all studies was calculated [39,40,41,42,43,44,47,49,50,51,52,53].

A total of 12 studies reporting associations between exposure and the outcome of interest were included in the graphical display of individual study estimates, with a summary estimate derived across studies. Individual study odds ratios (ORs) and corresponding 95% confidence intervals (CIs) are presented in the forest plot (Figure 2).

Across studies, point estimates were predominantly above unity, indicating a generally positive association, although effect sizes varied substantially. Reported ORs ranged from 0.66 (95% CI 0.43–0.91) to 4.30 (95% CI 1.20–15.00). Several studies demonstrated statistically significant associations, including those by Xie et al. [40] (OR 2.01, 95% CI 1.31–3.08), Berentzen et al. [42] (OR 1.85, 95% CI 1.07–3.21), Wright et al. [39] (OR 1.77, 95% CI 1.06–2.95), DeChristopher et al. [41] (OR 3.49, 95% CI 1.32–9.92), Reis et al. [50] (OR 1.83, 95% CI 1.22–2.76), Melo et al. [51] (OR 1.11, 95% CI 1.04–1.18), and Jeong et al. [53] (OR 1.07, 95% CI 1.03–1.12). In contrast, several studies reported estimates that crossed the null, including Scheffers et al. [43], Nagel et al. [47], and Silveira et al. [52].

An exploratory graphical summary of reported odds ratios suggested that many studies reported estimates above unity (OR 1.77, 95% CI 0.99–3.73); however, because of substantial heterogeneity in study design, exposure definitions, and outcome ascertainment, this estimate should not be interpreted as a pooled effect size or formal meta-analytic result. The forest plot was displayed on a logarithmic scale to accommodate the range of observed ORs and to facilitate comparison across studies and was included solely to illustrate the range and dispersion of reported associations across studies rather than to derive a quantitative pooled estimate.

## 4. Discussion

Current epidemiologic evidence suggests that high fructose exposure is associated with increased risk of pediatric asthma and, more selectively, with other atopic phenotypes, although effect sizes, exposed beverages, and outcomes vary across settings and designs; see Figure 2.

### 4.1. Strength and Consistency of Asthma Findings

The most consistent signal concerns asthma in relation to SSBs, EFF beverages, and selected fruit juices. Longitudinal cohorts (BRISA, PIAMA, Project Viva) report modest to moderate increases in asthma or asthma traits with higher early-life fructose or SSB intake, supporting temporality [37,38,39,42,43,48]. Large national datasets (NHANES, National Children’s Study) similarly show higher odds of current asthma with heavier SSB and EFF consumption, often approaching or exceeding two-fold [40,41,45]. Comparable associations are observed in a large California survey and in Brazilian (PeNSE) and Korean adolescent data linking soft drinks and ultra-processed dietary patterns to asthma and wheeze [50,51,53]. A Brazilian clinic-based study did not demonstrate a clear dose–response for severity, suggesting stronger associations with asthma occurrence than with symptom intensity 54.

Effect estimates range from modest (OR ~1.8 in Project Viva and for heavy soda intake in California) [39,50], to approximately two-fold in NHANES and PIAMA [40,42], and up to three- to five-fold in EFF-focused analyses combining HFCS soda, fruit drinks, and apple juice [41,45]. Ultra-processed pattern analyses generally report smaller but consistent associations (OR ~1.1–1.3) [51,53]. Heterogeneity likely reflects differences in fructose dose, beverage formulation, age at exposure, dietary context, and outcome definition. Nevertheless, the predominance of positive findings across independent populations, including analyses adjusted for adiposity and socioeconomic factors, suggests association, although findings remain heterogeneous across populations and study designs.

### 4.2. Role of Beverage Type and Fructose Formulation

Differentiating total SSBs from high-EFF beverages appears critical. Analyses from the National Children’s Study and US national data report particularly strong associations for beverages with fructose:glucose ratios > 1.0 (HFCS soda, fruit drinks, apple juice), with two- to five-fold higher asthma odds among frequent consumers [41,45]. This aligns with experimental evidence that unpaired fructose is more likely to escape absorption, undergo colonic fermentation, and promote barrier disruption and inflammatory signaling [14].

In contrast, ISAAC Phase Two and the French Six Cities study, using broad, non-differentiated beverage categories, found largely null associations [44,47], suggesting exposure misclassification may attenuate true effects. Fruit juice findings illustrate this heterogeneity: positive associations in PIAMA and among Brazilian Amazon schoolchildren [42,49], contrasted with neutral or inverse findings in ISAAC and French data [44,47]. Variability in juice composition, added sugars, fructose:glucose ratios, and cultural consumption patterns likely explains these discrepancies. Findings from the later PIAMA follow-up also suggested that associations were not consistent across adolescence [43].

### 4.3. Extension to Other Atopic Outcomes

Evidence for rhinitis, eczema, and sensitization is less consistent. BRISA reported positive associations between added sugars and early “allergy traits” [37,38], and Amazonian data linked frequent juice intake to active rhinitis 50. However, ISAAC and French Six Cities analyses found no robust associations with sensitization or eczema [44,47], and Korean adolescent data showed associations with asthma but not consistently with rhinitis or dermatitis [53]. Objective IgE or skin prick outcomes were infrequently assessed and mixed [44,46,47]. Overall, associations appear stronger and more reproducible for asthma and wheeze than for broader atopic phenotypes, suggesting fructose-related pathways may preferentially influence airway inflammation and hyperresponsiveness rather than systemic atopy.

### 4.4. Study Design, Measurement Error, and Residual Confounding

Interpretation is constrained by design limitations. Many large datasets are cross-sectional and rely on single dietary recalls or FFQs and self-reported asthma [40,41,44,45,47,50,51,53], limiting causal inference and raising potential for reverse causation. However, concordant findings in prospective cohorts (BRISA, PIAMA, Project Viva, puberty transition cohorts) mitigate this concern [37,38,39,42,43,48].

Exposure misclassification, particularly for EFF load and ultra-processed patterns, likely introduces nondifferential bias toward the null [40,41,50,51]. Outcome definitions also varied substantially [16,36,37,40,42,46,50,53]. Although most studies adjusted for BMI, socioeconomic status, and tobacco exposure [39,40,41,42,43,44,45,49,50,51,52,53], residual confounding remains plausible. Given this clinical and methodological heterogeneity, formal meta-analysis across all studies was not appropriate.

### 4.5. Integration with Mechanistic Evidence and Implications

Mechanistic data provide biological coherence. High fructose loads may exceed absorptive capacity, leading to malabsorption, dysbiosis, reduced short-chain fatty acid production, barrier dysfunction, endotoxemia, and activation of innate immune pathways (e.g., TLR4, RAGE, inflammasome signaling) [16,17,20,21,22,23,24,25,26]. Parallel metabolic effects, including uric acid generation, oxidative stress, and NLRP3 activation, may amplify systemic and airway inflammation [30].

Within this framework, the clustering of stronger epidemiologic associations around high-EFF beverages and early-life exposure is notable [37,38,39,42,43,45]. Longitudinal evidence linking increased SSB intake to greater post-exercise airway narrowing during puberty [48] further supports a life-course dimension. While causality cannot be definitively established, convergence of prospective data, cross-sectional consistency, biologic plausibility, and dose-relevant beverage profiles identify fructose exposure intake as a plausible exposure of interest that warrants further investigation in well-designed longitudinal studies (Supplementary Table S4).

Future cohorts should incorporate repeated, source-specific fructose quantification (including EFF load), objective biomarkers of gut permeability and systemic inflammation, and rigorous asthma phenotyping with lung function and endotype-relevant markers. Such integration is essential to clarify dose–response relationships, developmental windows, and interactions with adiposity, microbiome composition, and socioeconomic context, and to determine whether fructose reduction meaningfully reduces pediatric atopic disease burden.

## 5. Limitations

Several limitations of the review process should also be acknowledged. The search strategy was limited to two databases (PubMed and Embase), which may have reduced the sensitivity of the search. The review protocol was not prospectively registered, and screening and data extraction were conducted primarily by one reviewer with secondary verification rather than fully independent duplicate assessment. Finally, the graphical summary of effect estimates should not be interpreted as a formal meta-analysis.

## 6. Conclusions

High intake of fructose-containing beverages, particularly SSBs and excess free fructose sources such as apple juice and HFCS-sweetened drinks, is consistently, though heterogeneously, associated with increased risk of pediatric asthma, with less consistent links to other atopic outcomes. Null findings in large ISAAC datasets likely reflect substantial exposure misclassification.

Considered alongside mechanistic evidence implicating fructose malabsorption, gut barrier dysfunction, endotoxemia, and immunometabolic stress, the epidemiologic data suggest a biologically plausible but heterogeneous association. While causality remains unproven, fructose-rich beverages represent a plausible and potentially modifiable risk factor for pediatric atopic disease, warranting rigorous longitudinal studies with precise exposure and outcome assessment.

## Figures and Tables

**Table 1:** Characteristics and main findings of epidemiologic studies assessing fructose-containing beverages and pediatric atopic outcomes. Abbreviations: SSB, sugar-sweetened beverages; HFCS, high fructose corn syrup; EFF, excess free fructose; OR, odds ratio; CI, confidence interval; FFQ, food frequency questionnaire.

Full Title (First Author & Year)	Study Design & Setting	Sample Size & Population	Exposure Type	Exposure Assessment Method	Outcome Definition & Ascertainment	Statistical Models & Covariates	Effect Estimates (95% CI)	Quality Assessment (NOS)
Sugar-Sweetened Beverages and Allergy Traits at Second Year of Life: BRISA Cohort Study (Muniz et al., 2023) [37]	Birth cohort, São Luís BRISA (Brazil)	1144 children, age 2 years	Added sugars from SSBs (soft drinks, sweetened juices, chocolate milk)	24 h dietary recalls at 2 years	Latent “allergy traits” (atopic dermatitis, allergic rhinitis, food allergy diagnoses)	Structural equation modelling; adjusted for SES, sex, BMI z score, breastfeeding, infections	Standardized path coefficient for added sugars → allergy traits = 0.17 (*p* ≈ 0.025)	NOS: Moderate
Pathways in the Association Between Sugar Sweetened Beverages and Child Asthma Traits in the 2nd Year of Life: BRISA Cohort (Padilha et al., 2020) [38]	Birth cohort, BRISA (Brazil)	~1000–1100 children, age 2 years	SSB intake (soft drinks, sweetened juices)	24 h recalls at 2 years	Latent “asthma traits” (wheeze episodes, ER visits, asthma/rhinitis diagnosis)	Structural equation/path analysis; adjusted for BMI, SES, maternal factors	SSBs directly associated with higher asthma trait values (SC = 0.073; *p* = 0.030)	NOS: Moderate
Prenatal and Early Life Fructose, Fructose-Containing Beverages, and Midchildhood Asthma (Wright et al., 2018) [39]	Prospective birth cohort, USA	1068 mother–child pairs	Maternal SSBs, juices, total fructose in pregnancy; child fructose-containing drinks in early childhood	FFQs in 1st/2nd trimester and at median child age 3.3 years	Current asthma in mid-childhood (doctor diagnosed plus medication use or recent wheeze)	Logistic regression; adjusted for maternal BMI, smoking, SES, diet, child factors	OR 1.77 (95% CI 1.06–2.95) for higher early-childhood fructose intake	NOS: High
Association Between Asthma and Sugar-Sweetened Beverage Consumption in the United States Pediatric Population (Xie et al., 2021) [40]	Cross-sectional survey, NHANES (USA)	US children/adolescents 2–17 years (*n* ≈ 9000)	Total SSB energy intake (kcal/day)	24 h dietary recall	Current asthma (self-reported doctor diagnosis of asthma, still having asthma)	Survey-weighted logistic regression; adjusted for sex, age, race/ethnicity, BMI, SES, smoke exposure	Heavy SSB intake vs. non-SSB intake: aOR 2.01 (95% CI 1.31–3.08)	NOS: Moderate
Excess Free Fructose, Apple Juice, High Fructose Corn Syrup and Childhood Asthma Risk—the National Children’s Study (DeChristopher et al., 2020) [41]	Cross-sectional, US National Children’s Study cohort	~1900–2000 children	Excess free fructose via HFCS-soda, fruit drinks, apple juice	FFQ/recall for beverage frequency	Doctor-diagnosed asthma (parent report)	Logistic regression; adjusted for sex, BMI, energy intake and other confounders	Higher consumption associated with ~2–3.5 fold higher asthma odds (*p* ≈ 0.001 to <0.0001)	NOS: Moderate
Associations of Sugar-Containing Beverages with Asthma Prevalence in 11-Year-Old Children: The PIAMA Birth Cohort (Berentzen et al., 2015) [42]	Birth cohort, Netherlands (PIAMA)	2400–3300 children, age 11 years	100% fruit juice; sugar-added soft drinks; total sugar-containing beverages	Child-reported weekly glasses at 11 years	Asthma defined by wheeze, inhaled steroid use, and doctor diagnosis	Logistic regression; adjusted for sex, parental education, parental allergy, BMI, lifestyle	100% fruit juice ≥10 vs. ≤4 glasses/week: OR 2.09 (95% CI 1.21–3.60); sugar-added drinks: OR 1.56 (95% CI 0.95–2.56)	NOS: High
The Association of Pure Fruit Juice, Sugar-Sweetened Beverages and Fruit Consumption with Asthma Prevalence in Adolescents Growing up from 11 to 20 Years: The PIAMA Birth Cohort Study (Scheffers et al., 2022) [43]	Longitudinal birth cohort, Netherlands	>2000 adolescents, 11–20 years	Pure fruit juice, SSBs, fruit intake	Repeated FFQs (glasses/week or days/week)	Asthma prevalence at 11, 14, 17, 20 years (questionnaire-based)	GEE/logistic models; multivariable adjustment (diet, BMI, lifestyle)	Strongest association at 11 years: high fruit juice intake OR ≈ 1.8; no consistent association across 11–20 years	NOS: High
Prevalence and Association of Asthma and Allergic Sensitization with Dietary Factors in Schoolchildren: Data from the French Six Cities Study (Saadeh et al., 2015) [44]	Cross-sectional, multicenter, France	~50,000 children, 8–12 years	Multiple dietary items; juice and sweet beverages among them	Standardized ISAAC diet questionnaire	Asthma ever, current wheeze, allergic sensitization (SPT)	Random-effects meta-analytic logistic regression across centers	Fruit juice ≥3×/week vs. never: OR 0.66 (95% CI 0.43–0.91) for atopic wheeze; OR 0.73 (95% CI 0.55–0.96) for lifetime asthma	NOS: Moderate
Intakes of Apple Juice, Fruit Drinks and Soda Are Associated with Prevalent Asthma in US Children Aged 2–9 Years (DeChristopher et al., 2016) [45]	Cross-sectional, US national sample	1961 children aged 2–9 years	Apple juice, fruit drinks, HFCS-soda	Beverage frequency categories	Parent-reported doctor-diagnosed asthma	Logistic regression; adjusted for sex, BMI, energy intake	EFF beverages ≥5 vs. ≤1 time/month: OR 5.29; frequent apple juice ≥5 vs. ≤1 time/month: OR 2.43	NOS: Moderate
Dietary Intake in Adolescents with Asthma—Potential for Improvement (Bueso et al., 2011) [46]	Cross-sectional, clinic-based study, Norway	169 adolescents, 13–14 years (93 asthma, 76 controls)	SSBs, fruit juice, overall diet	FFQ; spirometry; skin prick testing	Asthma status (questionnaire + clinical assessment)	Logistic/linear models; adjusted for SES, BMI, lifestyle	Asthmatics showed higher sugared beverage intake; no single OR reported	NOS: Moderate
Effect of Diet on Asthma and Allergic Sensitisation in ISAAC Phase Two (Nagel et al., 2010) [47]	Multicountry cross-sectional, 29 centers, 20 countries	50,004 children, 8–12 years	Multiple foods; fruit juice and sweet drinks	ISAAC dietary frequency questionnaire	Wheeze, asthma ever, atopic sensitization	Random-effects meta-analysis of center-level logistic models	No association: fizzy drinks ≥3×/week vs. never, OR 0.96 wheeze; fruit juice ≥3×/week OR 0.91 wheeze	NOS: Moderate
The Potential Link Between Sugar-Sweetened Beverage Consumption and Post-Exercise Airway Narrowing Across Puberty (Emerson et al., 2016) [48]	Longitudinal cohort, puberty	*n* = 20	Habitual SSB intake	Diet questionnaire	Post-exercise airway narrowing (spirometry)	Longitudinal mixed models; adjusted for sex, BMI, activity	r = −0.61 post-pre puberty; r = −0.45 over time	NOS: Low (small sample)
Prevalence of Rhinitis and Associated Factors in Schoolchildren Who Live in the Amazon Islands (Freitas et al., 2016) [49]	Cross-sectional, Brazilian Amazon	400 children	Multiple dietary exposures including SSBs	Questionnaire	Rhinitis (and asthma) by ISAAC	Logistic regression; adjusted for BMI, SES, passive smoking	Fruit juice >2/week: OR 4.3 (95% CI 1.2–15.0)	NOS: Moderate
Dietary Factors Associated with Asthma Prevalence among Children in California (Reis et al., 2020) [50]	Birth-certificate–linked cross-sectional, California Health Interview Survey (USA)	56,312 children, 2–11 years	Energy-dense foods, soda/fast food (including SSBs)	Telephone diet questions, usual servings/week	Current asthma (parent-reported doctor diagnosis + “still has asthma”)	Survey-weighted logistic regression; adjusted for age, sex, race/ethnicity, BMI, SES, parental smoking	Soda ≥3 servings/day vs. none: OR 1.83 (95% CI 1.22–2.76)	NOS: Moderate
Associations of Ultra-Processed Food and Drink Products with Asthma and Wheezing among Brazilian Adolescents (Melo et al., 2018) [51]	Cross-sectional, nationally representative school survey (PeNSE 2012, Brazil)	109,104 adolescents, grades 9–12	Ultra-processed products (sweet biscuits, salty biscuits, ultra-processed meats, sweets/candies, soft drinks, packaged snacks)	Self-administered FFQ, days/week	Asthma ever, wheeze past 12 months (ISAAC)	Multivariable logistic regression; adjusted for age, sex, maternal education, region, activity, smoking, BMI	≥5 vs. 0–2 days/week: sweets/candies OR 1.08 (95% CI 1.03 to 1.13); soft drinks OR 1.14 (95% CI 1.05–1.22)	NOS: Moderate
Association between Dietary Habits and Asthma Severity in Children (Silveira et al., 2015) [52]	Cross-sectional outpatient/clinic-based, Brazil	Cases (*n* = 268) were children (3–12 yr) with persistent asthma and age-matched controls (*n* = 126) were those with intermittent asthma	Sweetened drinks, fast foods, snacks	FFQ/diet questionnaire	Asthma severity (symptoms, medications, GINA categories)	Ordinal/logistic regression; adjusted for age, sex, BMI, SES, activity, ETS	OR 0.88 (95% CI 0.57–1.36) highest vs. lowest intake	NOS: Moderate
Association of Soda Drinks and Fast Food with Allergic Diseases in Korean Adolescents (Jeong et al., 2024) [53]	Cross-sectional, nationally representative Korean Youth Risk Behavior Survey	865,614 Middle- and high-school adolescents	Soda drink consumption, fast food intake (times/week)	Self-administered questionnaire	Current asthma, allergic rhinitis, atopic dermatitis (self-reported doctor diagnosis)	Multivariable logistic regression was used to analyze the weighted odds ratios (ORs), along with 95% confidence intervals (CIs), for allergic diseases associated with the intake of soda drinks and fast food	Current asthma: soda OR 1.07 (95% CI 1.03–1.12), no consistent associations for rhinitis/AD	NOS: Moderate

## Data Availability

No new data were created or analyzed in this study.

## Supplementary Materials

The following supporting information can be downloaded at: https://www.mdpi.com/article/10.3390/nu18071057/s1, Table S1: Electronic Database Search Strategy for Fructose-Containing Beverages and Atopic Outcomes in Pediatric Populations. Table S2: Newcastle–Ottawa Scale (NOS) assessment of included observational studies. Table S3: PRISMA 2020 Checklist. Table S4: Synthesis of epidemiologic evidence linking fructose-containing dietary exposures with pediatric atopic outcomes.

## Abbreviations

The following abbreviations are used in this manuscript:

ATP	Adenosine Triphosphate
BMI	Body Mass Index
BRISA	Brazilian Ribeirão Preto and São Luís Birth Cohort Studies
EFF	Excess Free Fructose
FFQ	Food Frequency Questionnaire
HFCS	High Fructose Corn Syrup
IgE	Immunoglobulin E
IL-33	Interleukin 33
LPS	Lipopolysaccharide
NHANES	National Health and Nutrition Examination Survey
NOS	Newcastle–Ottawa Scale
OR	Odds Ratio
PIAMA	Prevention and Incidence of Asthma and Mite Allergy Birth Cohort
PRISMA	Preferred Reporting Items for Systematic Reviews and Meta-Analyses
SCFA	Short-Chain Fatty Acid
SSB	Sugar-Sweetened Beverage
TLR4	Toll-Like Receptor 4
VLDL	Very-Low-Density Lipoprotein
WHO	World Health Organization
ISAAC	International Study of Asthma and Allergies in Childhood
USDA	United States Department of Agriculture

## References

[1] García-Marcos L., Innes Asher M., Pearce N., Ellwood E., Bissell K., Chiang C.Y., El Sony A., Ellwood P., Marks G.B., Mortimer K. (2022). The burden of asthma, hay fever and eczema in children in 25 countries: GAN Phase I study. Eur. Respir. J..

[2] Oh J., Kim S., Kim M.S., Abate Y.H., Abd ElHafeez S., Abdelkader A., Abdi P., Abdulah D.M., Aboagye R.G., Abolhassani H. (2025). Global, regional, and national burden of asthma and atopic dermatitis, 1990–2021, and projections to 2050: A systematic analysis of the Global Burden of Disease Study 2021. Lancet Respir. Med..

[3] Lawson J.A., Brożek G., Shpakou A., Fedortsiv O., Vlaski E., Beridze V., Rennie D.C., Afanasieva A., Beridze S., Zejda J. (2017). An international comparison of asthma, wheeze, and breathing medication use among children. Respir. Med..

[4] Sarno G., Maio S., Stanisci I., Angino A., Tagliaferro S., Silvi P., Baldacci S., Sestini P., Pandics T., Hadjipanayis A. (2025). Asthma and allergies in schoolchildren: Data from the European SINPHONIE study. Int. J. Tuberc. Lung Dis..

[5] Brożek G., Lawson J., Shpakou A., Fedortsiv O., Hryshchuk L., Rennie D., Zejda J. (2016). Childhood asthma prevalence in Belarus, Ukraine, and Poland: BUPAS study. BMC Pulm. Med..

[6] Spolidoro G.C.I., Tesfaye Amera Y., Ali M.M., Nyassi S., Lisik D., Ioannidou A., Rovner G., Khaleva E., Venter C., van Ree R. (2022). Frequency of food allergy in Europe: An updated systematic review and meta-analysis. Allergy.

[7] Licari A., Magri P., De Silvestri A., Giannetti A., Indolfi C., Mori F., Marseglia G.L., Peroni D. (2023). Epidemiology of Allergic Rhinitis in Children: A Systematic Review and Meta-Analysis. J. Allergy Clin. Immunol. Pract..

[8] Gaillard E.A., Kuehni C.E., Turner S., Goutaki M., Holden K.A., de Jong C.C.M., Lex C., Lo D.K.H., Lucas J.S., Midulla F. (2021). Diagnosis of asthma in children aged 5–16 years: ERS guidelines. Eur. Respir. J..

[9] Wang S., Yin P., Yu L., Tian F., Chen W., Zhai Q. (2023). Effects of early diet on allergic diseases: A meta-analysis. Adv. Nutr..

[10] Asher M.I., Keil U., Anderson H.R., Beasley R., Crane J., Martinez F., Mitchell E., Pearce N., Sibbald B., Stewart A. (1995). International study of asthma and allergies in childhood (ISAAC): Rationale and methods. Eur. Respir. J..

[11] Acevedo N., Alashkar Alhamwe B., Caraballo L., Ding M., Ferrante A., Garn H., Garssen J., Hii C.S., Irvine J., Llinás-Caballero K. (2021). Perinatal and Early-Life Nutrition, Epigenetics, and Allergy. Nutrients.

[12] Zhang P. (2023). The Role of Diet and Nutrition in Allergic Diseases. Nutrients.

[13] Ma X., Nan F., Liang H., Shu P., Fan X., Song X., Hou Y., Zhang D. (2022). Excessive intake of sugar: An accomplice of inflammation. Front. Immunol..

[14] Yu J., Liu T., Guo Q., Wang Z., Chen Y., Dong Y. (2023). Disruption of the Intestinal Mucosal Barrier Induced by High Fructose and Restraint Stress Is Regulated by the Intestinal Microbiota and Microbiota Metabolites. Microbiol. Spectr..

[15] Merkiel S. (2014). Dietary intake in 6-year-old children from southern Poland: Part 1—Energy and macronutrient intakes. BMC Pediatr..

[16] Keshavarz F., Zeinalabedini M., Ebrahimpour-Koujan S., Azadbakht L. (2024). A systematic review and meta-analysis of the association of all types of beverages high in fructose with asthma in children and adolescents. BMC Nutr..

[17] Ferraris R.P. (2001). Dietary and developmental regulation of intestinal sugar transport. Biochem. J..

[18] Tappy L., Lê K.A. (2010). Metabolic Effects of Fructose and the Worldwide Increase in Obesity. Physiol. Rev..

[19] Douard V., Ferraris R.P. (2008). Regulation of the fructose transporter GLUT5 in health and disease. Am. J. Physiol.—Endocrinol. Metab..

[20] Simões C.D., Sousa A.S., Fernandes S., Sarmento A. (2025). Fructose Malabsorption, Gut Microbiota and Clinical Consequences: A Narrative Review of the Current Evidence. Life.

[21] Tsukamoto H., Takeuchi S., Kubota K., Kobayashi Y., Kozakai S., Ukai I., Shichiku A., Okubo M., Numasaki M., Kanemitsu Y. (2018). Lipopolysaccharide (LPS)-binding protein stimulates CD14-dependent Toll-like receptor 4 internalization and LPS-induced TBK1–IKKϵ–IRF3 axis activation. J. Biol. Chem..

[22] Frati F., Salvatori C., Incorvaia C., Bellucci A., Di Cara G., Marcucci F., Esposito S. (2018). The Role of the Microbiome in Asthma: The Gut–Lung Axis. Int. J. Mol. Sci..

[23] Dora D., Szőcs E., Soós Á., Halasy V., Somodi C., Mihucz A., Rostás M., Mógor F., Lohinai Z., Nagy N. (2024). From bench to bedside: An interdisciplinary journey through the gut-lung axis with insights into lung cancer and immunotherapy. Front. Immunol..

[24] Luo J., Ge X. (2025). Research Progress on Glycolysis in the Pathogenesis of Asthma. J. Asthma Allergy.

[25] Jaiswal N., Agrawal S., Agrawal A. (2019). High fructose-induced metabolic changes enhance inflammation in human dendritic cells. Clin. Exp. Immunol..

[26] Kabil A., Nayyar N., Brassard J., Li Y., Chopra S., Hughes M.R., McNagny K.M. (2024). Microbial intestinal dysbiosis drives long-term allergic susceptibility by sculpting an ILC2-B1 cell–innate IgE axis. J. Allergy Clin. Immunol..

[27] Ney L.M., Wipplinger M., Grossmann M., Engert N., Wegner V.D., Mosig A.S. (2023). Short chain fatty acids: Key regulators of the local and systemic immune response in inflammatory diseases and infections. Open Biol..

[28] Febbraio M.A., Karin M. (2021). “Sweet death”: Fructose as a metabolic toxin that targets the gut-liver axis. Cell Metab..

[29] Kelly R.S., Dahlin A., McGeachie M.J., Qiu W., Sordillo J., Wan E.S., Wu A.C., Lasky-Su J. (2017). Asthma Metabolomics and the Potential for Integrative Omics in Research and the Clinic. Chest.

[30] Singh S., Sharma A., Guru B., Ahmad S., Gulzar F., Kumar P., Ahmad I., Tamrakar A.K. (2022). Fructose-mediated NLRP3 activation induces inflammation and lipogenesis in adipose tissue. J. Nutr. Biochem..

[31] Geng Y., Faber K.N., de Meijer V.E., Blokzijl H., Moshage H. (2021). How does hepatic lipid accumulation lead to lipotoxicity in non-alcoholic fatty liver disease?. Hepatol. Int..

[32] Hernández-Díazcouder A., González-Ramírez J., Sanchez F., Leija-Martínez J.J., Martínez-Coronilla G., Amezcua-Guerra L.M., Sánchez-Muñoz F. (2022). Negative Effects of Chronic High Intake of Fructose on Lung Diseases. Nutrients.

[33] Lustig R.H. (2013). Fructose: It’s “Alcohol Without the Buzz”. Adv. Nutr..

[34] Reyes-Angel J., Kaviany P., Rastogi D., Forno E. (2022). Obesity-related asthma in children and adolescents. Lancet Child Adolesc. Health.

[35] Han M.W., Kim S.H., Oh I., Kim Y.H., Lee J. (2021). Obesity Can Contribute to Severe Persistent Allergic Rhinitis in Children Through Leptin and Interleukin-1β. Int. Arch. Allergy Immunol..

[36] Silverberg J.I., Kleiman E., Lev-Tov H., Silverberg N.B., Durkin H.G., Joks R., Smith-Norowitz T.A. (2011). Association between obesity and atopic dermatitis in childhood: A case-control study. J. Allergy Clin. Immunol..

[37] Muniz A.K.O.A., Vianna E.O., Padilha L.L., Nascimento J.X.P.T., Batista R.F.L., Barbieri M.A., Bettiol H., Ribeiro C.C.C. (2023). Sugar-Sweetened Beverages and Allergy Traits at Second Year of Life: BRISA Cohort Study. Nutrients.

[38] Padilha L.L., Vianna E.O., Vale A.T.M., Nascimento J.X.P.T., da Silva A.A.M., Ribeiro C.C.C. (2020). Pathways in the association between sugar sweetened beverages and child asthma traits in the 2nd year of life: Findings from the BRISA Cohort. Pediatr. Allergy Immunol..

[39] Wright L.S., Rifas-Shiman S.L., Oken E., Litonjua A.A., Gold D.R. (2018). Prenatal and Early Life Fructose, Fructose-Containing Beverages, and Midchildhood Asthma. Ann. Am. Thorac. Soc..

[40] Xie L., Atem F., Gelfand A., Delclos G., Messiah S.E. (2021). Association between asthma and sugar-sweetened beverage consumption in the United States pediatric population. J. Asthma.

[41] DeChristopher L.R., Tucker K.L. (2020). Excess free fructose, apple juice, high fructose corn syrup and childhood asthma risk—The National Children’s Study. Nutr. J..

[42] Berentzen N.E., van Stokkom V.L., Gehring U., Koppelman G.H., Schaap L.A., Smit H.A., Wijga A.H. (2015). Associations of sugar-containing beverages with asthma prevalence in 11-year-old children: The PIAMA birth cohort. Eur. J. Clin. Nutr..

[43] Scheffers F.R., Boer J.M.A., Gehring U., Koppelman G.H., Vonk J., Smit H.A., Verschuren W.M., Wijga A.H. (2022). The association of pure fruit juice, sugar-sweetened beverages and fruit consumption with asthma prevalence in adolescents growing up from 11 to 20 years: The PIAMA birth cohort study. Prev. Med. Rep..

[44] Saadeh D., Salameh P., Caillaud D., Charpin D., De Blay F., Kopferschmitt C., Lavaud F., Annesi-Maesano I., Baldi I., Raherison C. (2015). Prevalence and association of asthma and allergic sensitization with dietary factors in schoolchildren: Data from the french six cities study. BMC Public Health.

[45] DeChristopher L.R., Uribarri J., Tucker K.L. (2016). Intakes of apple juice, fruit drinks and soda are associated with prevalent asthma in US children aged 2–9 years. Public Health Nutr..

[46] Bueso A.K., Berntsen S., Mowinckel P., Andersen L.F., Lødrup Carlsen K.C., Carlsen K.H. (2011). Dietary intake in adolescents with asthma—Potential for improvement. Pediatr. Allergy Immunol..

[47] Nagel G., Weinmayr G., Kleiner A., Garcia-Marcos L., Strachan D.P. (2010). Effect of diet on asthma and allergic sensitisation in the International Study on Allergies and Asthma in Childhood (ISAAC) Phase Two. Thorax.

[48] Emerson S.R., Rosenkranz S.K., Rosenkranz R.R., Kurti S.P., Harms C.A. (2016). The potential link between sugar-sweetened beverage consumption and post-exercise airway narrowing across puberty: A longitudinal cohort study. Public Health Nutr..

[49] Freitas M.S., de Córdoba Lanza F., Monteiro J.C.S., Solé D. (2016). Prevalence of Rhinitis and Associated Factors in Schoolchildren who Live in the Amazon Islands. Am. J. Rhinol. Allergy.

[50] Reis W.P., Chai E., Gaio J., Becerra M.B., Banta J.E., Santos H.D. (2020). Dietary Factors Associated with Asthma Prevalence Among Children in California. Pediatr. Allergy Immunol. Pulmonol..

[51] Melo B., Rezende L., Machado P., Gouveia N., Levy R. (2018). Associations of ultra-processed food and drink products with asthma and wheezing among Brazilian adolescents. Pediatr. Allergy Immunol..

[52] Silveira D.H., Zhang L., Prietsch S.O.M., Vecchi A.A., Susin L.R.O. (2015). Association between dietary habits and asthma severity in children. Indian Pediatr..

[53] Jeong J., Jo H., Son Y., Lee S., Lee K., Choi Y., Lee H., Kim S., Jacob L., Smith L. (2024). Association of Soda Drinks and Fast Food with Allergic Diseases in Korean Adolescents: A Nationwide Representative Study. Int. Arch. Allergy Immunol..

