# Supplementary material for: Fructose-Containing Dietary Exposures and Pediatric Atopic Disease: A Review of Epidemiologic Evidence
Source: Nutrients. 2026 Mar 26;18(7):1057. doi: 10.3390/nu18071057 (PMC13074755; doi:10.3390/nu18071057)
Supplement: Supplementary file 1 [file nutrients-18-01057-s001.zip › nutrients-4210634-supplementary.pdf]

Supplementary Table S1: Electronic Database Search Strategy for Fructose-Containing Beverages and Atopic Outcomes in Pediatric Populations.

Database	Search Concept	Search Syntax
PubMed	Exposure Terms	("Fructose"[Mesh] OR fructose[tiab] OR "high fructose corn syrup"[tiab] OR HFCS[tiab] OR "sugar sweetened beverage*"[tiab] OR SSB[tiab] OR sucrose[tiab] OR "added sugar*"[tiab] OR "fruit juice"[tiab])
	Outcome Terms	("Asthma"[Mesh] OR asthma[tiab] OR wheeze*[tiab] OR "Dermatitis, Atopic"[Mesh] OR eczema[tiab] OR "atopic dermatitis"[tiab] OR "Rhinitis, Allergic"[Mesh] OR "allergic rhinitis"[tiab] OR "Food Hypersensitivity"[Mesh] OR "food allergy"[tiab] OR atopy[tiab] OR IgE[tiab])
	Population Terms	("Child"[Mesh] OR "Infant"[Mesh] OR "Adolescent"[Mesh] OR child*[tiab] OR infant*[tiab] OR toddler*[tiab] OR pediatric[tiab] OR paediatric[tiab] OR adolescent*[tiab])
	Filters Applied	Animal-only studies excluded using (animals[mh] NOT humans[mh])
Embase	Exposure Terms	('fructose'/exp OR fructose:ti,ab OR 'high fructose corn syrup'/exp OR 'high fructose corn syrup':ti,ab OR HFCS:ti,ab OR 'sugar sweetened beverage'/exp OR 'sugar-sweetened beverage*':ti,ab OR 'sugar sweetened beverage*':ti,ab OR SSB*:ti,ab OR 'soft drink'/exp OR 'soft drink*':ti,ab OR soda*:ti,ab OR 'fruit juice'/exp OR 'fruit juice*':ti,ab OR sucrose:ti,ab OR 'added sugar*':ti,ab)
	Outcome Terms	('asthma'/exp OR asthma:ti,ab OR wheeze*:ti,ab OR 'allergic rhinitis'/exp OR 'allergic rhinitis':ti,ab OR 'atopic dermatitis'/exp OR eczema:ti,ab OR 'atopic dermatitis':ti,ab OR 'food hypersensitivity'/exp OR 'food allergy':ti,ab OR atopy:ti,ab OR 'immunoglobulin e'/exp OR IgE:ti,ab)
	Population Terms	('child'/exp OR 'infant'/exp OR 'adolescent'/exp OR child*:ti,ab OR infant*:ti,ab OR toddler*:ti,ab OR pediatric*:ti,ab OR paediatric*:ti,ab OR adolescent*:ti,ab)
	Limits Applied	Publication years 2010–2025; Humans only

Supplementary Table S2: Newcastle–Ottawa Scale (NOS) assessment of included observational studies

Study	Study design	Representativeness of exposed cohort	Selection of non-exposed cohort	Exposure ascertainment	Outcome not present at start	Comparability (confounder control)	Outcome assessment	Follow-up adequacy	Total NOS score
Muniz et al., 2023 (BRISA)	Cohort	★	★	★	★	★★	★	★	8
Padilha et al., 2020 (BRISA pathways)	Cohort	★	★	★	★	★★	★	★	8
Wright et al., 2018 (Project Viva)	Cohort	★	★	★	★	★★	★	★	8
Berentzen et al., 2015 (PIAMA)	Cohort	★	★	★	★	★★	★	★	8
Scheffers et al., 2022 (PIAMA follow-up)	Cohort	★	★	★	★	★★	★	★	8

Study	Study design	Representativeness of exposed cohort	Selection of non-exposed cohort	Exposure ascertainment	Outcome not present at start	Comparability (confounder control)	Outcome assessment	Follow-up adequacy	Total NOS score
Emerson et al., 2015	Cohort	★	★	★	★	★	★	★	7
Xie et al., 2021 (NHANES)	Cross-sectional	★	—	★	—	★★	★	—	5
DeChristopher et al., 2020 (National Children's Study)	Cross-sectional	★	—	★	—	★★	★	—	5
DeChristopher et al., 2015	Cross-sectional	★	—	★	—	★★	★	—	5
Saadeh et al., 2015 (French Six Cities)	Cross-sectional	★	—	★	—	★★	★	—	5
Nagel et al., 2010 (ISAAC Phase II)	Cross-sectional	★	—	★	—	★★	★	—	5
Reis et al., 2020 (California Health Interview Survey)	Cross-sectional	★	—	★	—	★★	★	—	5
Melo et al., 2018 (PeNSE Brazil)	Cross-sectional	★	—	★	—	★★	★	—	5
Jeong et al., 2024 (Korean Youth Risk Behavior Survey)	Cross-sectional	★	—	★	—	★★	★	—	5
Silveira et al., 2015	Case-control / clinical sample	★	★	★	—	★	★	—	4
Freitas et al., 2016	Cross-sectional	★	—	★	—	★	★	—	4
Bueso et al., 2010	Cross-sectional	★	—	★	—	★	★	—	4

Note: Risk of bias for observational studies was assessed using the Newcastle–Ottawa Scale (NOS). The NOS evaluates three domains: selection of study groups, comparability of groups, and ascertainment of exposure or outcome. Maximum scores are nine stars for cohort studies and adapted scoring for cross-sectional studies.

Supplementary Table S3: PRISMA 2020 Checklist

Section	Item	PRISMA Checklist Item	Location in Manuscript
**TITLE**	1	Identify the report as a systematic review	Title page
**ABSTRACT**	2	Provide a structured summary including background, objectives, data sources, eligibility criteria, methods, results, limitations, and conclusions	Abstract
**INTRODUCTION**	3	Describe the rationale for the review in the context of existing knowledge	Introduction
	4	Provide an explicit statement of the objective(s) or question(s) the review addresses	End of Introduction
**METHODS**	5	Specify inclusion and exclusion criteria for the review and how studies were grouped for synthesis	Materials and Methods – Objective and Eligibility Criteria
	6	Specify all databases, registers, websites, organisations, reference lists, and other sources searched or consulted	Materials and Methods – Search strategy and data sources
	7	Present the full search strategies for all databases, including filters and limits used	Materials and Methods – Literature search strategy; Supplementary Table 1
	8	Specify the methods used to decide whether a study met the inclusion criteria	Materials and Methods – Study selection
	9	Specify the methods used to collect data from reports, including reviewer roles and consensus procedures	Materials and Methods – Data Extraction
	10	List and define all outcomes and other variables for which data were sought	Materials and Methods – Data Extraction
	11	Describe methods used to assess risk of bias in included studies	Materials and Methods – Quality Assessment
	12	Specify the effect measures used (e.g., odds ratios, risk ratios)	Materials and Methods – Data Extraction
	13	Describe methods used to synthesize results	Materials and Methods – Data Synthesis
	14	Describe methods used to explore heterogeneity (if applicable)	Narrative synthesis described in Data Synthesis
	15	Describe methods used to assess reporting bias (if applicable)	Not applicable – meta-analysis not performed
	16	Describe methods used to assess certainty of evidence	Risk of bias assessed using Newcastle–Ottawa Scale
**RESULTS**	17	Give numbers of studies screened, assessed for eligibility, and included in the review	Results; Figure 1 (PRISMA flow diagram)
	18	Present characteristics of included studies	Results; Table 1
	19	Present risk-of-bias assessments for each study	Results; Supplementary Table 2
	20	Present results of individual studies	Results; Table 1 and Figure 2
	21	Present results of syntheses	Results – Narrative synthesis
	22	Present results of investigations of heterogeneity	Results – Discussion of heterogeneity
	23	Present assessments of reporting bias	Not applicable – quantitative synthesis not conducted
**DISCUSSION**	24	Provide a general interpretation of the results in the context of other evidence	Discussion
	25	Discuss limitations of the evidence included in the review	Discussion – Study limitations
	26	Discuss limitations of the review process	Discussion – Limitations
	27	Discuss implications for practice, policy, and future research	Discussion and Conclusion
**OTHER INFORMATION**	28	Provide registration information for the review	Methods – protocol not prospectively registered
	29	Describe support or funding sources	Declarations – Funding
	30	Declare competing interests	Declarations – Declaration of interests
	31	Describe availability of data, code, or materials	Materials available within manuscript and supplementary tables

Supplementary Table S4: Synthesis of epidemiologic evidence linking fructose-containing dietary exposures with pediatric atopic outcomes. Legend: ●●●● = strong, consistent association across multiple studies, including higher-quality designs; ●●● = generally consistent association across several studies ●● = heterogeneous or mixed findings across studies; ● = limited evidence (few studies and/or imprecise estimates); ○

**Exposure Type**	**Asthma / Wheeze**	**Allergic Rhinitis**	**Atopic Dermatitis (Eczema)**	**Allergic Sensitization (IgE/SPT)**
**Sugar-Sweetened Beverages (SSBs)**	●●● Consistent positive association	● Limited positive evidence	● Limited positive evidence	○ Inconsistent or insufficient evidence
**High Excess-Free-Fructose (EFF) Beverages** ***(HFCS soda, fruit drinks, apple juice)***	●●●● Strong and consistent positive association	● Limited positive evidence	○ Insufficient evidence	○ Insufficient evidence
**Fruit Juice**	●● Heterogeneous findings	●● Heterogeneous findings	○ Insufficient evidence	○ Insufficient evidence
**Ultra-Processed Dietary Patterns / Sugary Foods**	●● Modest positive association	○ Insufficient evidence	○ Insufficient evidence	○ Insufficient evidence

= insufficient, null, or inconsistent evidence
